# GEROFIT: IMPACT ON PTSD SYMPTOMS AMONG PARTICIPATING VETERANS WITH SELF-REPORTED PTSD

**DOI:** 10.1093/geroni/igz038.133

**Published:** 2019-11-08

**Authors:** Cathy Lee, Rebecca Melrose, Erin Blanchard, Stacy Wilkins, Steven Castle, Katherine Hall, Miriam Morey

**Affiliations:** 1 VA Greater Los Angeles Healthcare System GRECC, Los Angeles, California, United States; 2 VA Greater Los Angeles Healthcare System/UCLA, Los Angeles, California, United States; 3 VA Greater Los Angeles Healthcare System, Los Angeles, California, United States; 4 Durham VA Healthcare System/Duke, Durham, North Carolina, United States

## Abstract

Post-traumatic stress disorder (PTSD) increases risk of medical comorbidities in aging. The Gerofit Program is an exercise program for older Veterans that shows efficacy for physical health. We sought to determine its impact on PTSD. Veterans in Gerofit completed a self-report questionnaire at 3 and 6 months assessing effect of Gerofit on: PTSD symptoms generally, disturbing dreams, avoidance, negative feelings, and irritability. Two hundred twenty-nine Veterans completed the questionnaire. Of these, 56 (24.5%) reported PTSD. None reported worsened PTSD following Gerofit participation. At 3 months, >50% of Veterans reported symptom improvement and this was maintained over 6 months for all items (p>0.05 paired t-test). There was an increase between 3 and 6 months in the percentage who reported “improved a lot” for overall symptoms (16.7% to 22.2%), negative feelings (5.6% to 11.1%) and irritability (0% to 11.1%). Gerofit may offer an effective intervention to improve PTSD symptoms in older Veterans.

